# Mechanical superiority of Pseudoxytenanthera bamboo for sustainable engineering solutions

**DOI:** 10.1038/s41598-023-45523-3

**Published:** 2023-10-24

**Authors:** N. Jiyas, Indu Sasidharan, K. Bindu Kumar, B. Gopakumar, Mathew Dan, B. Sabulal

**Affiliations:** 1 Department of Mechanical Engineering, Government Engineering College, Barton Hill, & APJ Abdul Kalam Technological University, Thiruvananthapuram, Kerala India; 2 Department of Chemistry, Government Engineering College, Barton Hill & APJ Abdul Kalam Technological University, Thiruvananthapuram, Kerala India; 3 Plant Genetic Resources, Jawaharlal Nehru Tropical Botanic Garden and Research Institute, Thiruvananthapuram, Kerala India; 4 Jawaharlal Nehru Tropical Botanic Garden and Research Institute, Thiruvananthapuram, Kerala India

**Keywords:** Mechanical engineering, Composites, Mechanical properties, Analytical chemistry, Imaging studies, Infrared spectroscopy, X-ray diffraction, Carbohydrate chemistry

## Abstract

The advancement in natural fibre composites has replaced synthetic fibres in various commercial sectors. Bamboo species possess high mechanical properties due to their lignocellulosic fibre content, which makes them suitable for engineering applications and potential alternatives to solid wood. However, despite Bamboo being composed of 130 genera and 1700 different species, out of which many still remains underexplored. In this study, we investigated the, Lignocellulosic profiling, fibre strength, and mechanical characterization of two species of Pseudoxytenanthera Bamboo: *Pseudoxytenanthera ritchiei*, *Pseudopxytenanthera stocksii*, and the results obtained were compared with *Bambusa balcooa*, one of the priority species of bamboo identified by The International Plant Genetic Resources Institute (IPGRI). BET (Brunauer–Emmett–Teller) was used to quantify the samples’ density, while SEM–EDX and FTIR spectroscopy were used for elemental analysis. The samples were then subjected to tensile test in addition, thermogravimetric analysis and water absorption test were carried out for the three species. The results showed that Pseudoxytenanthera species possessed superior chemical and mechanical characteristics compared to the priority species of bamboo used for composites. Out of the two Pseudoxytenanthera species studied, *Pseudoxytenanthera stocksii* exhibited the highest values of cellulose, hemicellulose, lignin, pectin, ash, carbon, and silicon, indicating its chemical superiority. Moreover, *Pseudoxytenanthera stocksii* also showed higher mechanical values for tensile strength, making it suitable for a variety of engineering applications. The TGA values also indicated that *Pseudoxytenanthera stocksii* is stable at high temperatures when compared with other natural fibres.

## Introduction

The growing concern over environmental challenges posed by the usage of non-renewable materials in various industries has spurred a shift towards renewable and sustainable alternatives. As the demand for materials continues to rise, there is a pressing need to explore efficient and eco-friendly solutions that can mitigate the adverse impacts of industrial processes^1^. While non-renewable resources still have substantial global reserves, the environmental consequences associated with their production and processing are becoming increasingly alarming, necessitating a focus on material efficiency and sustainability^2^. In response to these challenges, there has been a renewed interest in harnessing renewable resources^3–5^, with bamboo emerging as a promising nature-based solution (NbS). Bamboo offers several advantages over traditional wood and herbaceous biomass, such as rapid biomass accumulation, high photosynthesis rates, and exceptional carbon fixation capacity^6–8^. Additionally, lignocellulosic biomass from bamboo holds great promise, particularly in the development of nanocomposites, structural materials, and renewable energy applications^9,10^. The potential of bamboo as a superior renewable material has garnered attention, and several studies have focused on its composition and suitability for various applications. Notably, the bamboo species, with approximately 1700 reported species, has exhibited remarkable mechanical properties owing to its lignocellulosic fibre content, positioning it as a potential alternative to solid wood and synthetic fibres in commercial sectors^11^. Despite the significant potential, many bamboo species remain underexplored.

In this context, the present study investigates the taxonomy, Lignocellulosic profiling, fibre strength, and mechanical characterization of two varieties of Pseudoxytenanthera Bamboo species: *Pseudoxytenanthera ritchiei* and *Pseudoxytenanthera stocksii*. These species are compared with *Bambusa balcooa*, a priority bamboo species identified by The International Plant Genetic Resources Institute (IPGRI) for composite applications. The research aims to shed light on the potential superiority of Pseudoxytenanthera species in terms of chemical and mechanical characteristics when compared to widely used bamboo species^12,13^. To assess the chemical composition, density quantification was carried out using BET (Brunauer–Emmett–Teller) analysis, while elemental analysis was performed using SEM–EDX and FTIR spectroscopy. Furthermore, tensile tests were conducted to evaluate the mechanical properties of the three species. Additionally, thermogravimetric analysis (TGA) was employed to assess the thermal stability of the fibres, particularly focusing on *Pseudoxytenanthera stocksii*, to determine its suitability for high-temperature applications. The findings of this study hold significant potential for advancing the use of renewable and sustainable materials in various engineering applications. By identifying the superior characteristics of specific bamboo species, especially *Pseudoxytenanthera stocksii*, this research contributes valuable insights for the development of eco-friendly composites and construction materials, fostering a more sustainable future for industrial sectors.

## Materials and methods

### Material collection

The Jawaharlal Nehru Tropical Botanic Garden & Research Institute (JNTBGRI) is an R&D institution under Government of Kerala dedicated to the conservation and sustainable utilisation of India’s plant diversity, with a special focus on Kerala and the Western Ghats region. Among the various plant species conserved at JNTBGRI, bamboo holds significant importance in their conservation efforts. Three bamboo species, namely *Bambusa balcooa* (BB), *Pseudoxytenanthera ritchiei* (BR) and *Pseudoxytenanthera stocksii* (BS), have been selected for an extensive study. Specimens of these bamboo species were collected from the Bambusetum of JNTBGRI, which is located at latitude 8.7543° N and longitude 77.0247° E, at an altitude of 82 m above sea level. *Bambusa balcooa* (BB) and *Pseudoxytenanthera ritchiei* (BR) samples were identified by Dr. K C Koshy from Forest Research Institute Dehradun in 1998 and from Kerala Forest Research Institute Nilambur, Kerala in 1997 and conserved in JNTBGRI with voucher specimens kept in herbarium with accession no 43 and 64 respectively. Whereas *Pseudoxytenanthera stocksii* (BS) was identified from Machoor Mala, Kannur, Kerala in 2008 by Dr. B Gopakumar and conserved in JNTBGRI with voucher specimens kept in herbarium with accession no 891. The samples are stored in air tight containers. *Bambusa balcooa* has been chosen as the reference species due to its unique properties, including low density, high biodegradability, affordability, and impressive strength and stiffness. The study will compare the test results of the other bamboo species with those of *Bambusa balcooa*.

**Figure 1:**
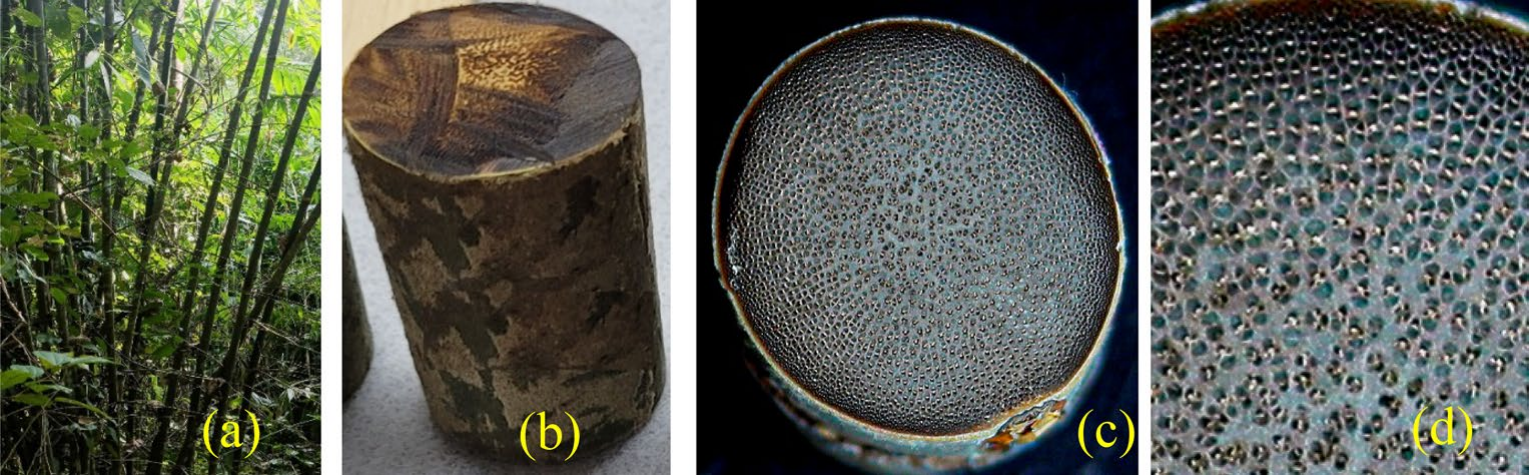
Images of the bamboo species—*Pseudoxytenanthera ritchiei* (**BR**).

Figure 1 illustrates *Pseudoxytenanthera ritchiei*^14,15^, an exquisite woody bamboo species found exclusively in the Western Ghats. These bamboos grow in clumps with a basal circumference of 2–5 m and reach a height of 3–8 m, with internodes measuring 30–40 cm at breast height. Figure 1a shows *Pseudoxytenanthera ritchiei* clumps and Fig. 1b shows longitudinal section of culm between the nodes. Each clump consists of 50–100 straight and solid culms, which are brownish green in colour and covered with a brown velvet tomentum when young. The poles of this bamboo are sturdy and upright, while the leaves are linear-lanceolate in shape. Figure 1c and d shows the aerial view of the cut section of culm detailing the fibre orientation for *Pseudoxytenanthera ritchiei*.

**Figure 2:**
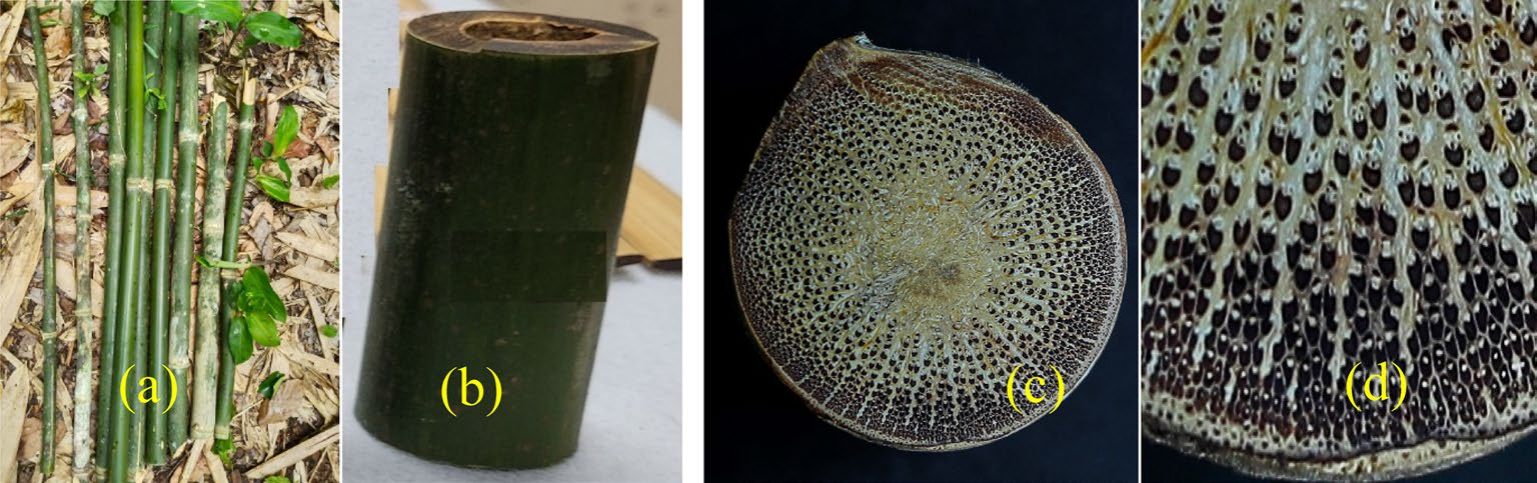
Images of the bamboo species—*Pseudoxytenanthera stocksii* (**BS**).

*Pseudoxytenanthera stocksii*^16–19^ is an impressive woody bamboo species shown in Fig. 2 known for its strength and resilience. These bamboos can reach a height of 10–15 m, with internodes measuring 30–40 cm at breast height. When young, the culms display a vibrant green colour with a distinctive greyish pubescence. Notably, the culm sheaths are adorned with brown persistent hairs, adding to the bamboo's unique appearance. Figure 2a shows *Pseudoxytenanthera stocksii* stems collected for the study and Fig. 2b shows longitudinal section of culm between the nodes. The leaves of *Pseudoxytenanthera stocksii* typically measure between 15 × 1.3 to 30 × 3 cm, showcasing a linear and lance-shaped structure. These features make *Pseudoxytenanthera stocksii* a valuable species, serving various purposes while contributing to the ecological significance and biodiversity of the Western Ghats, its native habitat. Figure 2c and d shows the top view of the cross section of culm detailing the fibre orientation *Pseudoxytenanthera stocksii*.

**Figure 3:**
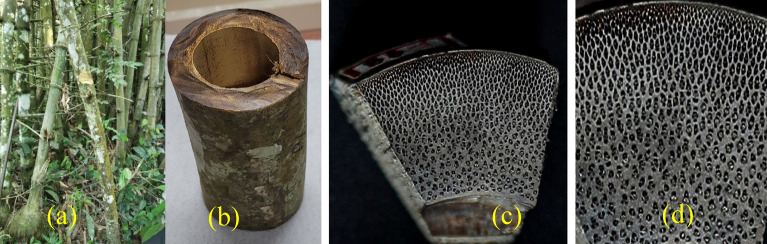
Images of the bamboo species—*Bambusa balcooa* (**BB**).

*Bambusa balcooa*^20^ shown in Fig. 3 is a remarkable drought resistant bamboo species with a unique ability to thrive in regions with low rainfall. This exceptional bamboo has the potential to yield over 100 metric tons per hectare, making it a valuable resource for sustainable cultivation and economic purposes. Originally native to the Indian subcontinent and Indo-China, *Bambusa balcooa* Roxb is a clumping bamboo known for its impressive height, reaching up to 25 m, and substantial wall thickness, measuring 15 cm. Figure 3a shows *Bambusa balcooa* culms and Fig. 2b shows longitudinal section of culm between the nodes Its ability to grow in arid conditions and its remarkable yield potential make it an essential species for conservation efforts and a promising option for various industries and applications. Figure 3c and d shows the top view of the cross section of culm detailing the fibre orientation *Bambusa balcooa*.

### Ethics statement

Experimental research and field studies on various species of bamboo reported here comply with relevant institutional, national, and international guidelines and legislation.

### Structures of the bamboo fibre

**Figure 4:**
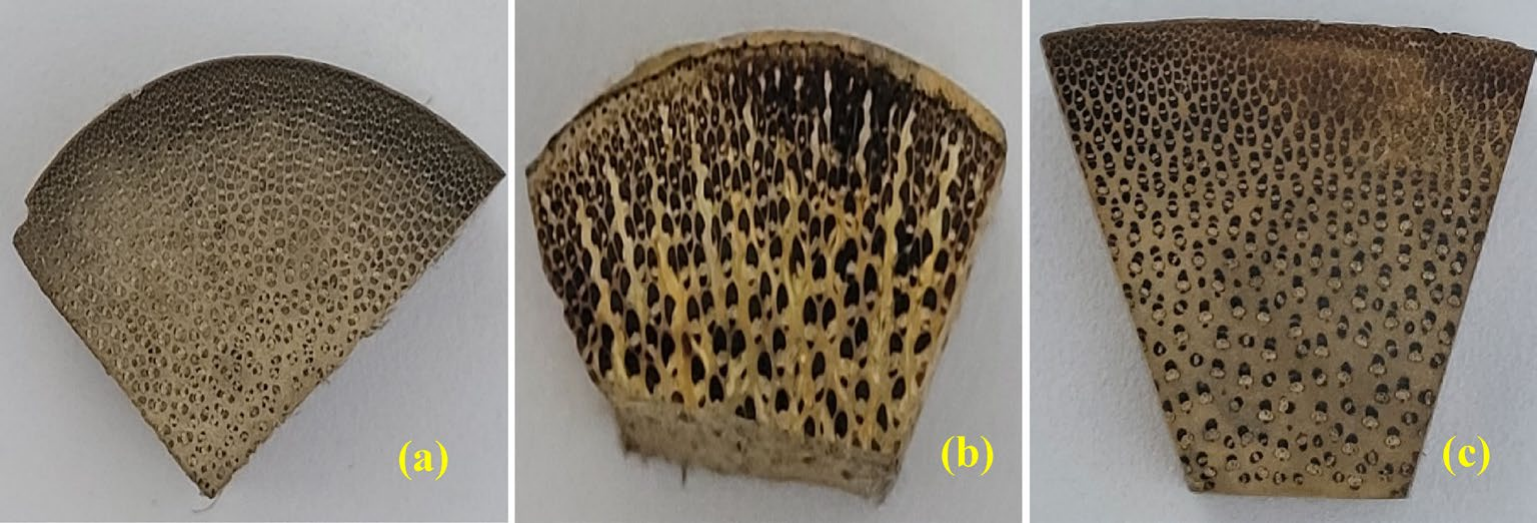
Fibre orientation in bamboo species.

Bamboo, a natural nanocomposite, boasts intricate structures at both macroscopic and microscopic levels. The arrangement of its fibres is such that they are densely packed near the outer boundary and sparsely distributed towards the center of the bamboo^21^ shown in Fig. 4. This unique design ensures consistent strength throughout its radial and lengthwise directions, as highlighted as suggested in literature^22^. Additionally, denser bamboo fibres exhibit alternating thick and thin lamellae, with higher lignin concentration found in the thinner lamellae compared to the thicker ones, as indicated by^23^. Studies by^24^ have revealed the main constituents of bamboo culms, which primarily consist of cellulose (60–70%), polysaccharides (20–25%), hemicelluloses, and lignin (each comprising approximately 20–30%). Furthermore, minor concentrations of resins, tannins, waxes, and inorganic salts are also present. The chemical composition of bamboo is comparable to that of hardwoods, with cellulose, hemicellulose, and lignin as its primary components. However, it differentiates itself by having higher alkaline extract, ash, and silica contents. Bamboo's multifaceted structure and chemical composition make it an exceptional natural material with versatile applications. Its unique fibre arrangement ensures excellent mechanical properties, providing strength and flexibility. The abundance of cellulose offers significant potential for various industrial uses, particularly in the production of paper, textiles, and bioenergy. Additionally, bamboo's high lignin content makes it durable and resistant to decay, making it suitable for construction and outdoor applications. The diverse properties of bamboo, along with its sustainability and renewability, have led to increasing interest in utilizing it as a green alternative in various industries. As a renewable resource, bamboo plays a crucial role in sustainable development and environmental conservation efforts. With ongoing research and advancements in processing technologies, bamboo's potential is expected to be further unlocked, enabling a wider range of innovative and eco-friendly applications across different sectors.

**Figure 5:**
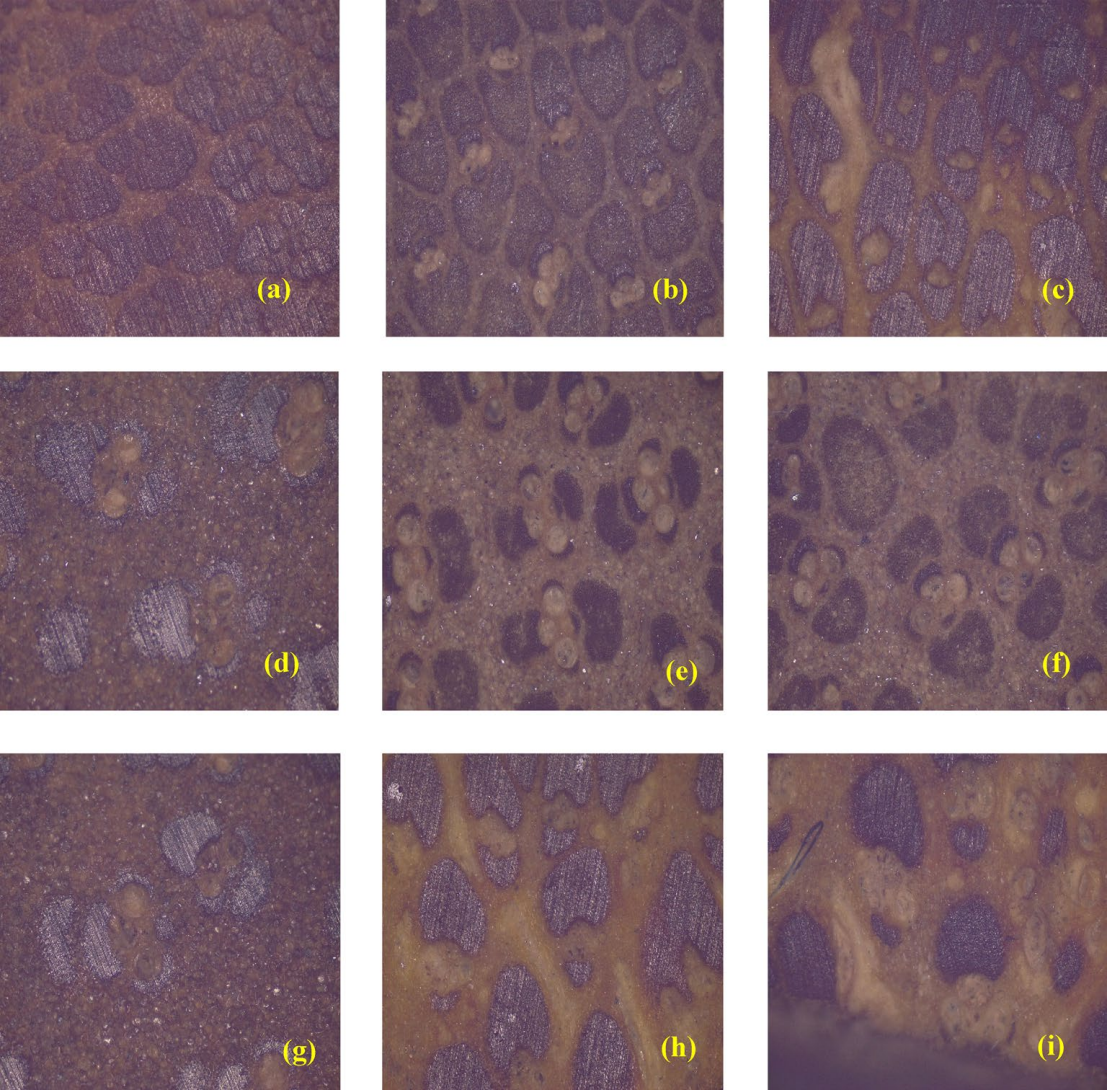
Optical microscopic images of cross section of bamboo culms.

From Fig. 4a–c shows the cross-sectional images of *Pseudoxytenanthera ritchiei*, *Pseudoxytenanthera stocksii* and *Bambusa balcooa* respectively obtained using digital camera, it is evident that the fibres are closely packed towards the outer portion of the culm. For detailed analysis, the specimens were taken to LEIKA Optical Stereo Microscope and images obtained are shown below. The optical microscopic images reiterate the fact that the culm is denser as it progresses outwards radially and the matrix is predominant as it reaches the centre of culm radially. Figure 5a, d and g corresponds to *Bambusa balcooa* images from the radially outer portion to the centre respectively, similar cases for Fig. 5b, e and h for *Pseudoxytenanthera ritchiei* and Fig. 5c, f and i for *Pseudoxytenanthera stocksii*. From these images it is evident that *Pseudoxytenanthera ritchiei* has fibres are densely populated compared with other two.

### Fibre extraction

As we traverse from the inner to the outer region of the bamboo stem, a consistent pattern of increasing tensile strength in the bamboo fibres becomes evident. Moreover, there is a noticeable rise in the tensile elastic modulus, while the strain to failure decreases. The fibres closer to the inner part exhibit a brittle fracture behaviour, while those nearer to the outer part demonstrate characteristics similar to ductile fracture^25^. In this particular study, the focus was on extracting fibres from the outer sheath of the bamboo stem. Three common methods for bamboo fibre extraction are available: mechanical, chemical, and a combination of both^26^. For this research, the mechanical extraction method called mechanical retting was employed. The process involved obtaining fresh internode billets from carefully selected bamboo plants and cutting them into 200 mm sections. Each section was further divided into six equally sized strips along the grain. These strips were soaked in water for approximately three days to facilitate the extraction process. Subsequently, the researchers manually scraped the bamboo fibre bundles from the soaked strips using a sharp knife. Although the fibres broke along their length during this process, they were found to be of strong and good quality. Only fibre bundles longer than 80 mm, possessing even thickness and a smooth surface, were selected to create tensile specimens. A total of 150 specimens were prepared, divided into six groups, each comprising 25 specimens. To ensure consistent moisture content and avoid any potential moisture-related variability in results, all the specimens were placed in a constant temperature and humidity box set at 20 °C and 65% relative humidity for moisture equilibration. This meticulous preparation and extraction process aimed to obtain reliable and standardized tensile specimens for further testing and analysis. The mechanical retting method was chosen due to its effectiveness in preserving the integrity and strength of the extracted fibres. By employing such carefully controlled procedures, the researchers sought to explore the mechanical properties of the bamboo fibres comprehensively, providing valuable insights into their potential applications in various industries, including construction, textiles, and engineering. Additionally, understanding the varying mechanical behaviour of the fibres along the stem could shed light on the structural design and engineering applications of bamboo-based materials, which are increasingly recognized for their sustainability and eco-friendliness.

#### Determination of bamboo fibre yield

Bamboo fibres were isolated by the chemical retting process^27^. Oven dried bamboo samples were cut into strips (20 × 2 × 2 mm), and immersed in the chemical solution containing 30% H_2_O_2_, Distilled water and Glacial acetic acid in the ratio 1:4:5 at a temperature of 60 °C for 42 h. The extracted fibres were then isolated by filtration and they were dried in oven at 75 °C for overnight. The yield of fibre extraction was calculated as a

$$Yield \left( \% \right) = \frac{Fibre\; weight\left( g \right)}{{Weight\; of \;Bamboo \;strips \left( g \right) }} \times 100$$

The fibre yield obtained for all the three species is 63.2% for *Pseudoxytenanthera ritchiei* and 65.27% for *Pseudoxytenanthera stocksii*, and 63.26 for *Bambusa balcooa*.

### Physical characterization

#### Diameter and Density

The diameter of Bamboo fibres was determined by measuring three random locations of 100 fibres using an optical microscope with specific specifications. Longitudinal direction images were captured from distinct fibre samples. The mean diameter of the Bamboo fibre was then calculated using the software “Image Pro Plus. ” To assess the density of Bamboo fibres, a pycnometer setup was used. In this setup, the fibre density was identified by comparing it with the known density of toluene (${\rho }_{t}$ = 0.866 g/cm^3^).

$${\varvec{\rho}} = \user2{ }\frac{{\left( {{\varvec{m}}_{2} - {\varvec{m}}_{1} } \right)}}{{\left( {{\varvec{m}}_{3} - {\varvec{m}}_{1} } \right) - \left( {{\varvec{m}}_{4} - {\varvec{m}}_{2} } \right)}} \times {\varvec{\rho}}_{{\varvec{t}}}$$

where, $\rho$ and ${\rho }_{t}$ express the density of bamboo fibres and toluene respectively in g/cm^3^. Also ${{\varvec{m}}}_{1}$*, *${{\varvec{m}}}_{2}$*,* ${{\varvec{m}}}_{3}$ and ${{\varvec{m}}}_{4}$ mass of the empty pycnometer, mass of the pycnometer filled with chopped fibres, mass of the pycnometer filled with toluene, and mass of the pycnometer filled with fibres and toluene in gram, respectively. The density of the Bamboo fibre was determined using a specific equation^25^. By employing these methods, researchers were able to accurately measure the diameter and density of bamboo fibres^26,28^. These parameters are essential for understanding the fibre's physical characteristics and its potential applications in various fields, including textiles, composites, and other engineering materials.

### Chemical characterization

#### Determination of cellulose, hemicellulose & lignin

The cellulose, hemicellulose, and lignin content of the samples were determined^29^. First, the sample was refluxed with ethanol four times, with each reflux lasting for 15 min. It was then washed with distilled water and dried in an oven at 40 °C overnight to estimate the dry weight (A). Then the sample was treated with 24% KOH (Potassium hydroxide) for 4 h at 25 °C. Afterwards, it was thoroughly washed with distilled water and dried in an oven at 80 °C overnight, and the dry weight (B) was measured. The sample was treated with 72% H_2_SO_4_ (Sulphuric acid) for 3 h to hydrolyse the cellulose, and then refluxed with 5% H_2_SO_4_ for 2 h. The samples were washed with distilled water and dried in an oven at 80 °C overnight, and the dry weight (C) was measured. By following this method, the cellulose, hemicellulose, and lignin content of the samples could be determined accurately as follows.

$$Cellulose = B - C,\;Hemicellulose = A - B\;and\;Lignin = C.$$

The series of treatments allowed for the isolation and quantification of each component separately. Cellulose is the main component responsible for the structural strength of plant cell walls, while hemicellulose contributes to the amorphous regions of the cell wall and plays a role in bonding cellulose fibres together. Lignin, on the other hand, provides rigidity and resistance to degradation. Understanding the content of these components is crucial for assessing the quality and potential applications of the plant materials being studied.

#### Moisture content

Strictly adhering to ASTM D 2495 standards, the moisture content percentage of the specimens was measured by using the oven drying method^30^. The moisture content in the Bamboo fibres was determined by equation^31, 32^.

$$\user2{M }\left( \user2{\% } \right) = \frac{{{\varvec{W}}_{1} - {\varvec{W}}_{2} }}{{{\varvec{W}}_{1} }} \times {\mathbf{100}}$$

where, M (%) denotes the percentage of moisture content, W_1_ and W_2_ are the fibre weight before and after drying in grams.

#### Water absorption

An adaptation of the ASTM D570-98 standard was employed to assess water absorption in the test specimens. In this modified approach, the specimens were submerged in distilled water maintained at 21.5 °C for varying time spans, with a maximum of 60 min. The samples were retrieved from the water at specific intervals: 1, 3, 5, 10, 15, 20, 25, 30, and 60 min. Once removed, the samples were rapidly rid of any surface dampness and then weighed with precision to the nearest 0.0001 g. The difference in weight post-immersion was used to quantify the absorbed water volume. This methodology offers insights into the material's water uptake kinetics and sheds light on how it responds to prolonged exposure to water under controlled conditions.

$${\text{Water}}\;{\text{ Absorption }}\left( \% \right) \, = \frac{{{\text{weight }}\;{\text{after }}\;{\text{immersion }} - {\text{ weight }}\;{\text{before}}\;{\text{ immersion }}}}{{{\text{weight }}\;{\text{before }}\;{\text{immersion}}}} \times 100\%$$

### FTIR analysis

The Infrared spectroscopic analysis of the fibres were carried out using the FTIR-IR Prestige-21 (Schimadzu Corporation, Japan)^3^. The powdered bamboo fibres were mixed Potassium bromide powder (ratio 1:10) then formed into a pellet for analysis. The graph was plotted between the percentage of transmitted IR light and the wave number (4000–400/cm).

### X‑ray diffraction (XRD)

The crystallinity index of the bamboo fibre specimen was studied using X‑ray diffraction (XRD) technique. The degree of structural arrangement is determined through the crystallinity index which is important in determining the mechanical properties of natural cellulosic fibres^33^. Fibres with high crystallinity index or cellulose content have been found to possess superior mechanical properties. The XRD study was carried at room temperature using an X-ray diffractometer (Rigaku, Japan) out on using Cu–K_α_ source with wavelength 1.54 Å. 0.5 g of the samples was mounted on the sample holder. The X-Ray detector offered in the diffractometer was used to scan the diffracted X-ray between 0° and 40° (2θ) at a scan speed 4°/min in steps of 0.02°. The crystallinity index (CI) of bamboo fibre was then determined using empirical methods^34^ as presented in Equation.

$$CI\left( \% \right) = \frac{{I_{200} - I_{am} }}{{I_{200} }} \times 100$$

where, I_200_ is the maximum intensity of the peak between 22° and 23° for the crystalline area at a 2θ angle, and *I*_*am*_ is the minimum intensity of an amorphous region between 18° and 19° at a 2θ angle. The crystallite size (CS) of the fibre was determined by the Scherrer’s equation as given below,

$$CS = \frac{K\lambda }{{\beta cos\theta }}$$

where, K denotes Scherrer’s constant, whose value is 0.89 and, α, β & ϴ are wavelength of the radiation, full-width at half-maximum, Bragg angle, respectively.

### Thermo-gravimetric analysis (TGA)

The thermal stability of bamboo fibres was assessed through Thermogravimetric Analysis (TGA) using a Perkin Elmer TGA 4000 instrument. Approximately 10 mg of bamboo fibre powders were accurately weighed and subjected to controlled temperature ramping. The weight changes of the fibres were recorded throughout the analysis. The experiments were conducted under a nitrogen atmosphere, with a constant heating rate of 10 °C per minute and a flow rate of 30 ml per minute, ranging from room temperature up to 600 °C^35^. TGA analysis provides crucial insights into the thermal stability and decomposition behaviour of bamboo fibres. By observing the weight changes at different temperatures, valuable information about the fibre’s degradation characteristics can be obtained. Additionally, Broido's equation and plot were utilized to determine the Kinetic Activation Energy (E_a_) of the bamboo fibres. The kinetic activation energy represents the minimum amount of energy required for the fibre to degrade. This information is significant for understanding the thermal performance and potential applications of bamboo fibres, especially in industries where heat resistance and stability are essential factors. By evaluating the thermal properties of bamboo fibres, researchers can further optimize their usage in various fields, including composites, textiles, and construction materials.

$$\ln \left[ {\ln \left( \frac{1}{y} \right)} \right] = - \left( {\frac{{E_{a} }}{R}} \right)\left[ {\left( \frac{1}{T} \right) + K} \right]$$

where, ‘y’ represents the normalized weight of the sample (w_t_/w_i_), where ‘w_t_’ denotes the weight of the sample at any time ‘t’, and ‘w_i_’ represents the initial weight of the sample. The variable ‘T’ represents the temperature at the specific time under consideration, while 'R' represents the universal gas constant with a value of 8.32 J/mol K. This notation and representation are used to analyse the weight changes and thermal behaviour of the sample over time, particularly in the context of thermogravimetric analysis (TGA). The use of ‘y’ as a normalized weight allows for direct comparison and understanding of the sample's degradation and decomposition behaviour during the temperature ramping process. By employing such standardized notation, researchers can assess the kinetics and activation energy associated with the sample’s thermal stability, providing valuable insights into its behaviour and potential applications.

### Differential scanning calorimeter analysis

To complement the thermogravimetric analysis, Differential Scanning Calorimetry (DSC)^34^ was conducted to investigate the thermal transitions of bamboo. The DSC experiments were carried out using a Perkin Elmer DSC 6000 instrument. Samples weighing 20 mg were placed in closed aluminium pans for analysis. The protocol included three heating runs, two cooling runs, and isothermal annealing at 160 °C for durations ranging from 10 to 120 min. This annealing process induced physical aging in the material, leading to an increase in the aging overshoot. The primary objective of the DSC analysis was to determine the glass transition temperature (T_g_) by observing the aging behaviour. The measurements were conducted between 50 and 250 °C, utilizing a constant heating rate of 20 °C per minute under a nitrogen flow. This method allowed researchers to gain valuable insights into the thermal behaviour and transitions of bamboo, particularly in the context of glass transition temperature determination. By performing DSC in conjunction with thermogravimetric analysis, a comprehensive understanding of bamboo’s thermal characteristics and stability could be obtained, facilitating its potential applications in various industries, such as plastics, composites, and other thermally sensitive materials.

### Morphological study by SEM analysis

The surface morphology of the three sample fibres was examined using scanning electron microscopy at an operating voltage of 10 kV using JEOL JSM-IT510 instrument. To enhance the conductivity of the fibre before the experiment, the samples were coated with gold in a vacuum environment.

### EDX analysis

Energy dispersive X-ray spectroscopy or EDX is a popular technique for identifying the elemental components of natural fibre like Carbon, Oxygen, and Nitrogen. The elemental composition of bamboo fibres was determined with EDX JEOL JSM-IT510 instrument associated with the SEM.

### BET adsorption measurements

Bamboo fibre surface characteristics, such as specific surface area, constant C, and pore volume, were assessed using the BET (Brunauer Emmett and Teller) analysis with a specific instrument. The total pore volume was determined from isotherm plots of nitrogen adsorption at various relative pressures (0.02–1 kPa) and a constant temperature of 350.3 °C. Prior to the analysis, impurities in the fibres were removed through a degassing process involving heat, vacuum, and flowing gas. This allowed for a thorough evaluation of the fibres’ surface properties, vital for understanding their potential applications in adsorption, catalysis, and other relevant fields.

### Tensile strength

Tests were performed using a Computerized Universal Testing Machine, specifically the KALPAK UTM, Model No. KIC-2-1000-C, with a maximum load capacity of 100 kN and a 0.1 kN load cell, to determine the tensile strength of individual bamboo fibres. The tests followed the ASTM D3822-07 standard, treating bamboo fibres similarly to natural and textile fibres. Various fibre lengths were examined during the experiments to obtain comprehensive data on their tensile properties.

## Results and discussion

### Physical characterization

#### Density

**Figure 6:**
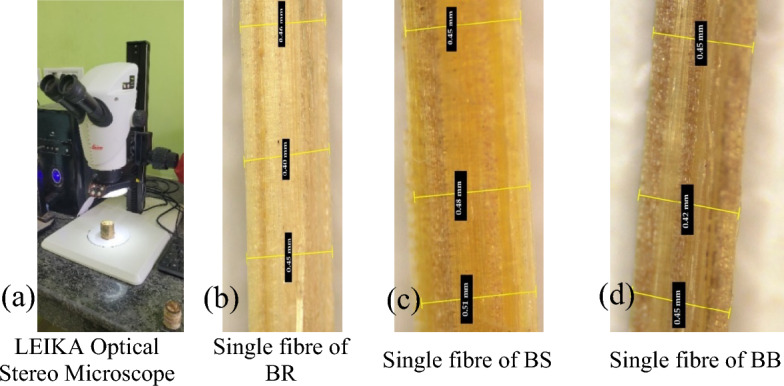
Diameter measurements of single bamboo fibre using Optical Stereo Microscope.

The physical characterization of bamboo fibres was done by thickness/diameter and density measurements. Like any other natural fibre, the bamboo fibres are of uneven thickness hence the diameter measurement was carried out at different lengths of the fibres shown in Fig. 6. The analysis using optical microscope indicated that all the bamboo fibres individually measured, the diameter varied from 0.3 to 0.4 mm and the mean diameter was found to be 0.35 mm. Figure 6 shows the diameter measurement and microscopic view of a fibre. The density value of the Bamboo fibres ranged from 1.08 to 1.24 g/cc. The species Pseudoxytenanthera ritchie showed the highest value for density measurement. Density measurements are always an important factor in assessing the mechanical property since there fibres are of natural origin^6^. The density of the bamboo fibre is comparable to the other economically important fibres^36^. The diameter and density values were given in Table 1.

**Table 1:** Physical and chemical characterization of bamboo fibres-experimental results.

Sample	Diameter (mm)	Density (g/cm^3^)	Moisture (weight %)	Cellulose (%)	Hemicellulose (%)	Lignin (%)
*Bambusa balcooa* (BB)	0.3–0.4 mm	1.14	8.84	60.98	23.0	16.06
*Pseudoxytenanthera ritchiei* (BR)	0.3–0.4 mm	1.24	4.80	75.05	3.1	21.85
*Pseudoxytenanthera stocksii* (BS)	0.3–0.4 mm	1.08	4.04	72.93	8.3	18.82

Experimental setup for the measurement of fibre diameter using LEIKA Optical Stereo Microscope make is shown in Fig. 6a, diameter measurements of single fibres for Pseudoxytenanthera ritchiei (BR), Pseudoxytenanthera stocksii (BS) and Bambusa balcooa (BB) are shown in Fig. 6b–d respectively.

### Chemical characterization

#### Determination of cellulose, hemicellulose and lignin

The cellulose, hemicellulose and lignin content of the samples were determined using the method mentioned earlier. The two varieties BR and BS possessed very high cellulose content of 75.05% and 72.93% respectively compared to the common variety BB (60.98%). It is considered that the high cellulose content in plant fibres increases the tensile strength, stability, stiffness and hydrolysis resistance which are the important parameters in commercialization of the fibres^37^. The high cellulose content of the samples in present study is the best indication of the superior quality of these two varieties. The hemicellulose content varies from 3.1 to 23% which also attributes to the high tensile strength of the fibres. The lignin content of the two varieties were 21.85% (BR) and 18.82% (BS). A previous study on different bamboo varieties shows that lignin content varies from 16 to 25%^38^. The values were tabulated in Table 1.

#### Moisture content

A 5-g fibre sample was collected under standard atmospheric conditions for testing at 20 °C and 65% relative humidity. These weighted fibre samples were then positioned in a consistent-temperature air oven set at 105 °C. At 15-min intervals, the sample weight was recorded until the change in mass between consecutive weighing was less than 0.1%. By comparing the initial standard conditioning weight with the weight after oven-drying, the moisture content in individual bamboo fibres was calculated using the oven drying method. The recorded moisture content for bamboo fibres ranged from 4.04 to 8.84%.

#### Water absorption

The outcomes of the water absorption test, conducted according to the earlier-described method, are tabulated and are shown in the Table 2 below.

**Table 2:** Percentage water absorption of Bamboo fibres-experimental results.

Sample	Time (min)								
	1	3	5	10	15	20	25	30	60
*Bambusa balcooa* (BB)	43.86	58.24	65.52	71.32	74.46	77.16	80.61	83.73	95.6
*Pseudoxytenanthera ritchiei* (BR)	31.79	46.98	52.56	57.15	61.06	64.57	66.08	69.96	85.9
*Pseudoxytenanthera stocksii* (BS)	28	39.52	44.09	51.3	56.82	60.22	62.52	65.2	82.72

The investigation underscores disparities in fibre density across different varieties. Particularly noteworthy is *Bambusa balcooa*, which exhibits heightened water absorption in comparison to the other species. In contrast, *Pseudoxytenanthera stocksii* demonstrates remarkably reduced water absorption. This variance can be traced back to distinctive surface characteristics, as evidenced by SEM images. *Bambusa balcooa* reveals a higher count of surface pores as evident from morphological analysis and also the higher pore volume as evident from BET results in Table 7, contributing to its higher water absorption capability when compared to the other species. On the contrary, *Pseudoxytenanthera stocksii* follows an opposing pattern, showcasing fewer surface pores coupled with low pore volume as evident from BET results in Table 7. These findings offer insights into the relationship between surface features and water absorption tendencies in these bamboo varieties.

#### FTIR analysis

**Figure 7:**
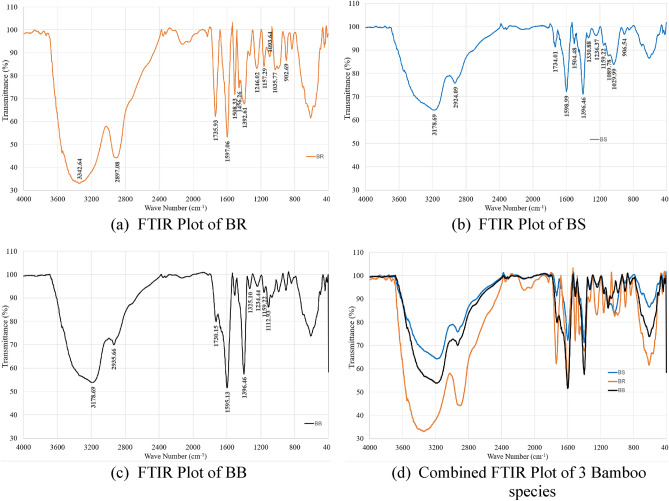
FTIR Plots of bamboo fibres of different Species.

Fourier-Transform Infrared Spectroscopy (FTIR) was carried out on all the three varieties of the bamboo fibres and results obtained are plotted as shown in Fig. 7. Figure 7a–c corresponds to FTIR spectrum for, *Pseudoxytenanthera ritchiei* (BR), *Pseudoxytenanthera stocksii* (BS) and *Bambusa balcooa* (BB) respectively and Fig. 7d is intended for a comparison between all three varieties. The spectrum shows the functional groups present in the different components present in the fibres.

The spectrum shows an absorbance band corresponding to phenolic hydroxyl, alkenic, aromatic and beta glycosidic groups since the main constituents of the fibres were cellulose, hemicellulose, and lignin. The different absorbance frequencies and their corresponding functional groups are shown in Table 3. The various peaks in the spectrum were assigned based on the earlier reports^39^. All the three samples showed similar spectrum with significant peaks at ~ 3344/cm (phenolic –OH– stretching), ~ 2923/cm (C–H stretching), ~ 1598 & ~ 897/cm (β-glycosidic linkage characteristics of lignin).

**Table 3:** FTIR analysis—assignment of absorbance peaks.

Wavenumber (/cm)			Vibration and functional group	Corresponding component
BR	BS	BB		
3344	3180	3196	–O–H stretching	Cellulose, Hemicellulose and lignin
2902	2929	2931	–CH_2_ and –CH stretching	Cellulose, Hemicellulose and lignin
1737	1734	1728	C=O stretching	Hemicellulose
1597	1598	1598	Aromatic ring vibration	Lignin
1508	1504	1504	Aromatic skeletal vibrations	Lignin
1458	1448		–OH, in plane bending	Cellulose, Hemicellulose and lignin
1388	1402	1398	CH bending	Cellulose and Hemicellulose
	1328	1330	CH_2_ wagging	Cellulose
1249	1242	1238	C–O stretch and OH in plane	Polysaccharides
1163	1151	1163	C–O–C Asymmetric stretching	
	1095	1109	C–O–C stretching and Aromatic C–H plane deformation	Cellulose and Lignin
1029	1031	1068	C–O stretching	Holocellulose and Lignin

#### X-ray diffraction analysis

**Figure 8:**
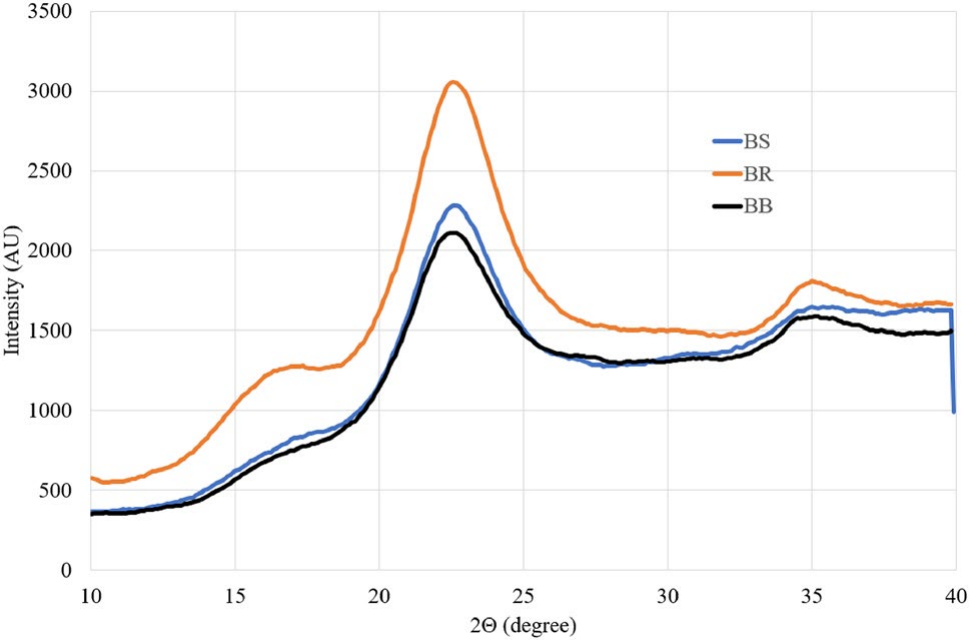
X-ray diffraction pattern of fibres of different bamboo species.

The X-ray diffraction pattern of the three bamboo fibre samples are shown in Fig. 8. The spectrum shows three significant peaks at 2θ values approximately 16.15°, 22.46° and 35.46° corresponding to the lattice planes (200), (110) and (004). The main peak at 22.46° is considered to be that of cellulose^33^. The cellulose crystallinity index ranges from 45 to 65% which is higher than other commercially available natural fibres^40,41^. The more crystallinity of cellulose, the less hemicellulose present^42^, which is evident from our chemical characterization study. Also, the crystallite structure of bamboo fibre estimated from the study is 24–26 Å range. It is known that low crystallite structure reduces the water absorption and chemical reactivity when used in composite^43^_._ These three bamboo fibres are therefore a good contender for the natural composites industry due to their extremely low crystallite structure values recorded in Table 4. Increased elongation, moisture recovery, and chemical reactivity of the fibre are all associated with decreased crystallinity and higher proportion of amorphous cellulose^44^.

**Table 4:** Cellulose crystallinity index and size of fibres of different bamboo species.

Sample	Crystallinity index (%)	Crystallite size (Å)
BR	60.08	26.12
BS	65.56	24.22
BB	45.04	25.37

#### Thermo gravimetric analysis

**Figure 9:**
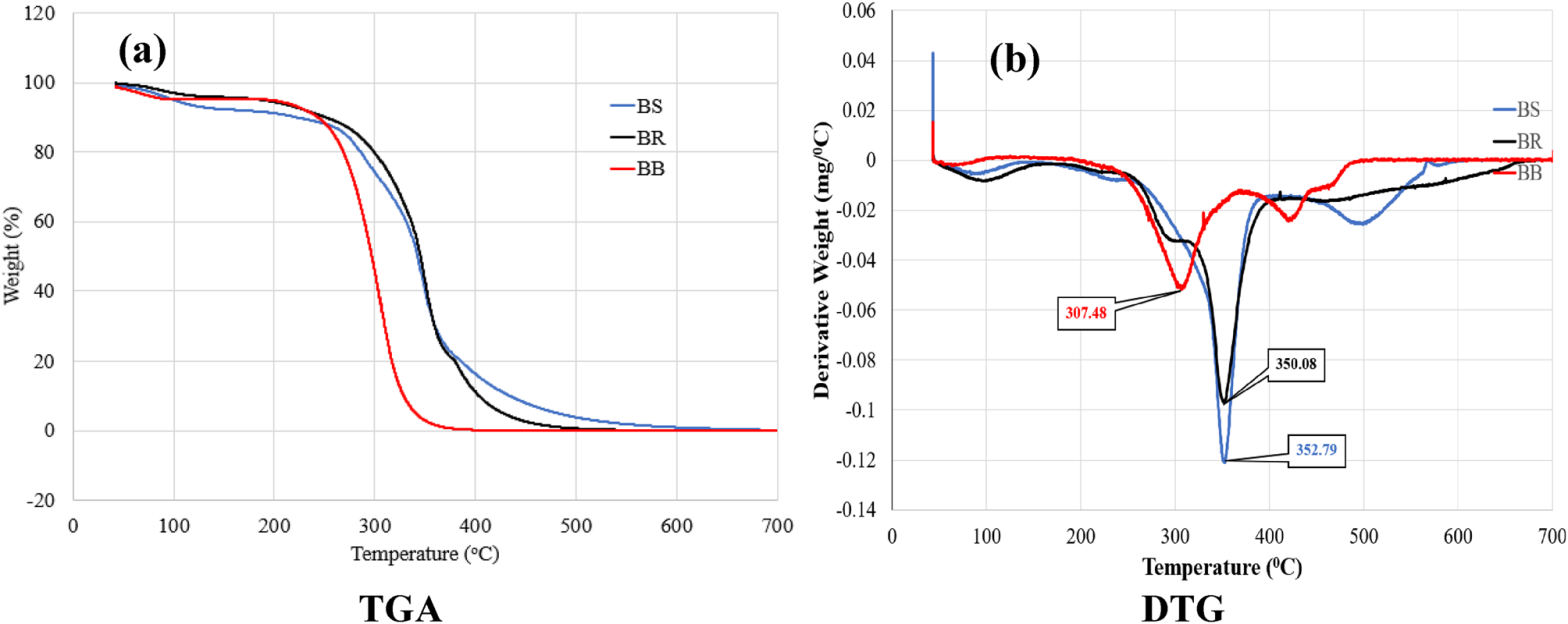
(**a**) Plots of TGA, (**b**) DTG curves of fibres of different species.

Thermal degradation behaviour of the bamboo fibres was analysed by TGA curves and the results were shown in Fig. 9. The thermograms demonstrated mainly three stage degradation. In stage-I (25–150 °C), weight loss occurred due to evaporation of moisture. The amount of moisture in a bamboo culm varies between species, in individual culms within same species and in different parts along the same bamboo culm and this resulted in different weight losses during this stage. The weight loss % ranged from 1.75 (BB) to 1.43 (BR). In stage II (250–300 °C), loss of hemicellulose occurs. In the case of the ritchie since the hemicellulose content is very low confirmed by the chemical analysis, the hemicellulose degradation is not visible in the thermogram. This incurred loss of weight from 18.57% (*BB*) to 3.5% (*BR)*. In stage III (300–350 °C), primarily thermal degradation of cellulose occurred^45^ resulting massive loss of weight, which ranged from 33% (BS), 41.2% (BB) and 65% (BR). After that slow degradation of lignin occurred until end^46^. With respect to thermal stability, *Bambusa balcooa* was the least stable species demonstrating maximum degradation at 304.60 °C (Table 5, Fig. 9a), and *Pseudoxytenanthera stocksii* is the most stable one (T_max_ = 349 °C).

**Table 5:** Recorded values of decomposition temperature from TGA results.

Sample	Moisture content (%)	Maximum decomposition temperature, T_max_ (°C)
BR	8.84	350.08
BS	4.80	352.79
BB	4.04	307.48

#### Differential scanning calorimeter (DSC) analysis

**Figure 10:**
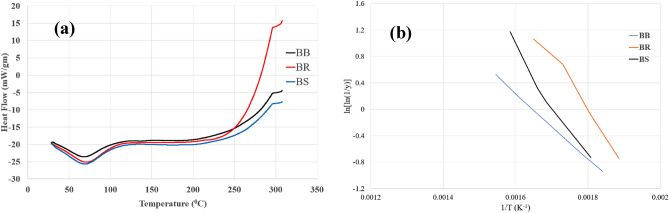
Plots of (**a**) DSC Curves, (**b**) Broido’s plot of fibres of three species of bamboo.

Utilizing Differential Scanning Calorimetry (DSC) as shown in Fig. 10a, transitions within bamboo fibres were examined. DSC thermograms were captured within the temperature range of fibre thermal stability, determined by the onset temperature from TGA curves. Among all three samples, a notable endothermic occurrence around 70 °C was evident, attributed to water desorption—a phenomenon supported by previous research^20,39^. Following this, no further exothermic or endothermic behaviors were detected. This analysis provides insights into the thermal characteristics of bamboo fibres and highlights the water desorption process as a significant transition within the examined temperature range.

The investigation into thermal stability using Thermogravimetric Analysis yielded thermal decomposition data as shown in Fig. 9b. These values were employed to calculate the activation energy for three types of fibres using the Broido’s plot as shown in Fig. 10b: *Bambusa balcooa* displayed an activation energy of 41.3 kJ/mol, *Pseudoxytenanthera ritchiei* exhibited 65.6 kJ/mol, and *Pseudoxytenanthera stocksii* demonstrated an activation energy of 69.3 kJ/mol. This analysis offers valuable insights into the energy required for thermal decomposition in these distinct fibre varieties, contributing to a deeper understanding of their thermal properties and potential applications.

#### Scanning electron microscopy

**Figure 11:**
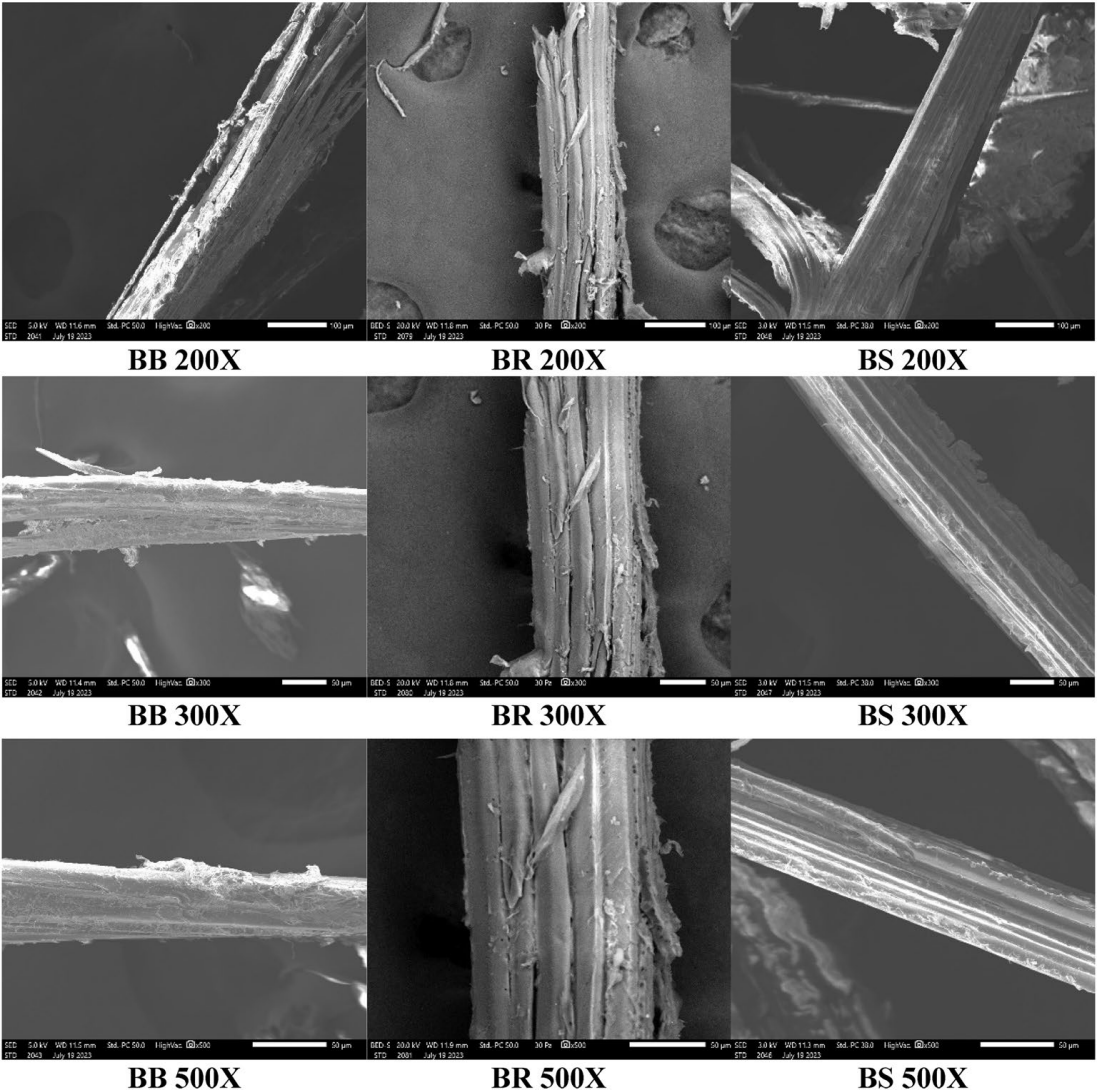
SEM Images of bamboo fibres of different species.

The surface morphologies of all three species of bamboo fibres used in this study were analysed by Scanning Electronic Microscopic (SEM) (JEOL JSM-IT510) at an operating voltage 10 kV. Before the experiment, the samples were coated with gold in a vacuum atmosphere to improve the conductivity of fibre. The images obtained for various magnification are shown in Fig. 11.

SEM was employed to analyze the surface morphology of Bamboo fibres. The surface exhibited an uneven texture and harbored organic components and contaminants. The imagery revealed that the fibres possessed a circular cross-section and were adorned with microfibrils running along their length (refer to Fig. 11). Evidently, the surface was tainted with substances like waxes, dirt, and oils. In order to render these natural fibres suitable for composite fabrication, it's imperative to have a rough surface devoid of waxes and impurities. Thus, a chemical pre-treatment is required for these sampled fibres.

#### Energy dispersive X-ray spectroscopy (EDX)

EDX is a common method used to identify elements (for instant Carbon, Oxygen, and Nitrogen etc.) of natural fibre. The elemental presence of bamboo fibre was determined by EDX (JEOL JSM-IT510), which is equipped with the SEM.

**Figure 12:**
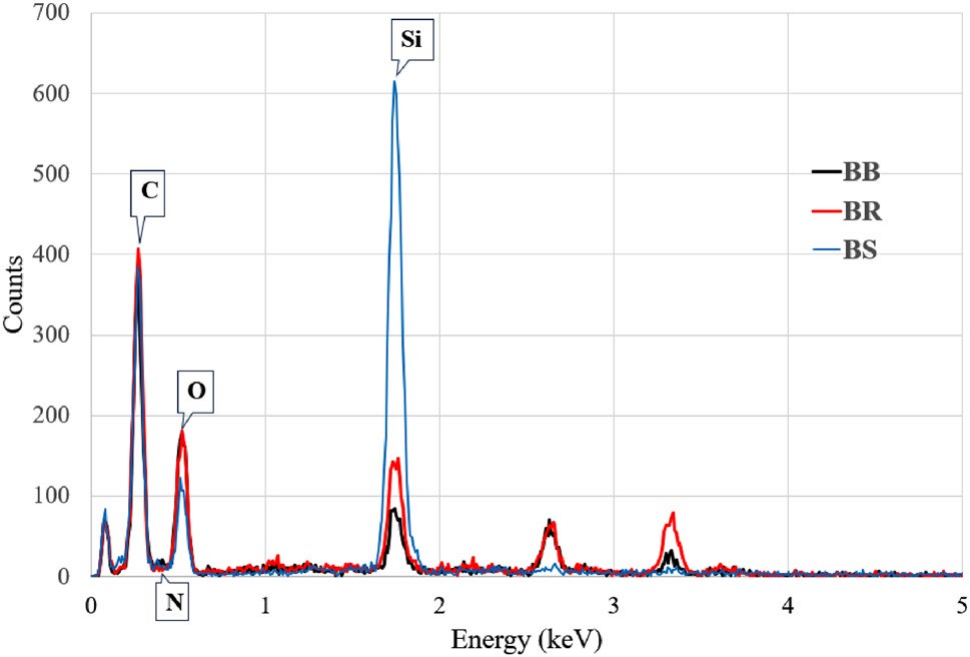
EDX Plot of bamboo fibres of different species.

The quantitative elemental analysis of the bamboo fibres were shown in Fig. 12 in terms of weight and atomic percentage. The basic elements in the bamboo fibres are carbon and oxygen, which are the major elements on the fibre surface shown in Table 6.

**Table 6:** Elemental composition of bamboo fibres from SEM–EDX analysis.

Element	BR		BS		BB	
	Weight %	Atomic %	Weight %	Atomic %	Weight %	Atomic %
Carbon (C)	57.71	64.85	57.99	67.54	51.93	59.12
Nitrogen (N)	5.44	5.29	9.67	9.66	8.5	8.36
Oxygen (O)	31.99	27.24	17.78	15.54	36.11	30.86
Silicon (Si)	5.4	2.62	14.56	7.25	3.41	1.66

#### BET adsorption

The BET (Brunauer Emmett and Teller) adsorption isotherm is a model used to describe the adsorption of gas molecules on to solid surfaces. It is commonly applied to study the adsorption behaviour of porous materials, including bamboo fibres. The BET isotherm helps to understand the specific surface area and adsorption capacity of the fibres. The larger surface area in turn results in a greater number of contact sites between consecutive fibres. This enhances the utility of the scaffold as a bio-adsorbent for waste water treatment^47^.

Results of BET analysis of the three sample fibres were recorded in Table 7. The comparison of BET results shows that all the three isotherms are nearly same and which looks like isotherms of other natural fibres such as flax, Hemp and sisal^48^. The specific surface area of the BR sample is higher compared to other two (Table 7) implies that BR is having higher hydrophobicity^49^.

**Table 7:** Recorded values of pore volume and surface area of bamboo fibres from BET Analysis.

Sample	Specific surface area (m^2^/g)	C constant	Micropore volume (cm^3^/g)
BR	1.835	7.60317	0.004
BS	1.128	7.40768	0.003
BB	1.315	8.72204	0.004

## Tensile test

**Figure 13:**
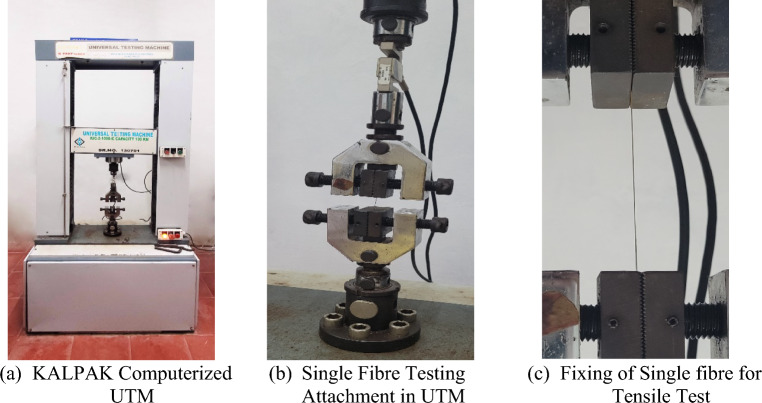
Tensile test set up for Single fibre testing.

As stated above tensile tests were performed using the KALPAK UTM, Model No. KIC-2-1000-C, (Fig. 13) with a maximum load capacity of 100 kN and a 0.5 kN load cell, by following the ASTM D3822-07 standard. Prior to conducting the tensile testing, a thorough visual inspection of the bamboo fibres was carried out to ensure their integrity and absence of any defects along their length. To ensure secure gripping and proper alignment between the clamps, both ends of the fibre samples were attached to a paper tab using an appropriate adhesive. Throughout the entire handling process, great care was taken to prevent the introduction of any additional defects and to preserve the original distribution of flaws in the fibres. The length of the tensile test samples was determined by the size of the opening in the paper frame, ensuring consistency in the test conditions for accurate comparisons.

In this study, researchers investigated the tensile properties of single fibres from three different species using a universal testing machine and are recorded the results in Table 8.

**Table 8:** Experimental values of Tensile test results of different species of bamboo fibres.

Fibre length	Strength parameters	Bamboo varieties		
		*Bambusa balcooa*	*Pseudoxytenanthera ritchiei*	*Pseudoxytenanthera stocksii*
20 mm	Peak load (N)	40.50	52.00	35.00
	Stress (N/mm^2^)	345.56	475.66	415.78
	Strain	0.035	0.045	0.03
	E (GPa)	33.00	32.53	28.00
40 mm	Peak load (N)	36.30	36.00	26.20
	Stress (N/mm^2^)	325.58	425.66	380.25
	Strain	0.021	0.018	0.018
	E (GPa)	18.28	24.00	20.18
60 mm	Peak load (N)	28.50	35.50	30.00
	Stress (N/mm^2^)	301.65	389.55	365.77
	Strain	0.012	0.011	0.010
	E (GPa)	29.64	30.03	28.00
80 mm	Peak load (N)	35.50	34.00	50.00
	Stress (N/mm^2^)	281.75	375.66	345.83
	Strain	0.01	0.0081	0.0138
	E (GPa)	24.30	28.31	31.33

Throughout the experiments, they deliberately varied the gauge length, which refers to the tested fibre’s length. Notably, a consistent trend was observed across all three species: as the gauge length increased, the strength of the fibres decreased. This behavior can be attributed to variations in the cross-sectional area, as the fibre diameters ranged from 0.3 to 0.4 mm and failure occurred at the minimum cross-sectional area^22^.

The recorded maximum tensile stress values were 345.56 N/mm^2^, 475.66 N/mm^2^, and 415.78 N/mm^2^ for *Bambusa balcooa*, *Pseudoxytenanthera ritchiei*, and *Pseudoxytenanthera stocksii*, respectively, at the minimum gauge length of 20 mm. These results clearly indicate that the *Pseudoxytenanthera* species of bamboo exhibits higher strength compared to the conventionally used *Bambusa balcooa* species. Thus, the tensile properties of the fibres are significantly influenced by both their gauge length and species, with *Pseudoxytenanthera* species showing superior strength characteristics in this study.

## Conclusion

The work reported here focused on two *Pseudoxytenanthera* bamboo species, *Pseudoxytenanthera ritchiei* and *Pseudoxytenanthera stocksii*, comparing them with *Bambusa balcooa*, a priority bamboo species for composite applications. Results reveal that *Pseudoxytenanthera* species, especially *Pseudoxytenanthera stocksii*, possess superior chemical and mechanical properties. *Pseudoxytenanthera stocksii* exhibits higher cellulose, hemicellulose, lignin, and pectin content, making it a chemical standout. Additionally, it demonstrates greater tensile strength and thermal stability, making it suitable for high-temperature applications. These findings underscore the potential of different bamboo species, particularly *Pseudoxytenanthera stocksii*, in eco-friendly composites and construction materials, promoting sustainability in industrial sectors.

## Data Availability

Correspondence and requests for materials should be addressed to the corresponding author.
